# Supplementary material for: Single-cell RNA-seq reveals hidden transcriptional variation in malaria parasites
Source: eLife. 2018 Mar 27;7:e33105. doi: 10.7554/eLife.33105 (PMC5871331; doi:10.7554/eLife.33105)
Supplement: Transparent reporting form [file elife-33105-transrepform.pdf]

eLIFE
1st Floor
24 Hills Road
Cambridge CB2 1JP, UK
P 01223 855340
W elifesciences.org
T @elife

eLife's transparent reporting form

We encourage authors to provide detailed information *within their submission* to facilitate the interpretation and replication of experiments. Authors can upload supporting documentation to indicate the use of appropriate reporting guidelines for health-related research (see EQUATOR Network), life science research (see the BioSharing Information Resource), or the ARRIVE guidelines for reporting work involving animal research. Where applicable, authors should refer to any relevant reporting standards documents in this form.

If you have any questions, please consult our Journal Policies and/or contact us: editorial@elifesciences.org.

Sample-size estimation

- You should state whether an appropriate sample size was computed when the study was being designed
- You should state the statistical method of sample size computation and any required assumptions
- If no explicit power analysis was used, you should describe how you decided what sample (replicate) size (number) to use

Please outline where this information can be found within the submission (e.g., sections or figure legends), or explain why this information doesn't apply to your submission:

We did not perform power analyses for our experiments. These were exploratory analyses, the first of their kind for parasites, and we did not know what the size of the signal would be. At a bulk level, typically 3-6 samples per treatment provide power to distinguish highly differentially expressed genes between treatments using RNAseq. As we were exploring single cells of potentially different life cycle stages, we aimed to have at least 10 cells per life stage (indeed, we had many more than this for most life stages). We achieved this for all life stages apart from P. falciparum males, which we therefore did not characterize more deeply.

Replicates

- You should report how often each experiment was performed
- You should include a definition of biological versus technical replication
- The data obtained should be provided and sufficient information should be provided to indicate the number of independent biological and/or technical replicates
- If you encountered any outliers, you should describe how these were handled
- Criteria for exclusion/inclusion of data should be clearly stated
- High-throughput sequence data should be uploaded before submission, with a private link for reviewers provided (these are available from both GEO and ArrayExpress)

Please outline where this information can be found within the submission (e.g., sections or figure legends), or explain why this information doesn't apply to your submission:

eLife Sciences Publications, Ltd is a limited liability non-profit non-stock corporation incorporated in the State of Delaware, USA, with company number 5030732, and is registered in the UK with company number FC030576 and branch number BR015634 at the address 1st Floor, 24 Hills Road, Cambridge CB2 1JP | August 2014

Descriptions of the experiments are provided in the methods section entitled "Sequencing of single-cell libraries".

Biological replication has two meanings in our work. Firstly, cells of a particular cell type/stage are biological replicates of each other for the purposes of determining variability in gene expression in that cell type. Secondly, experiments on P. falciparum and P. berghei are in a sense biological replicates to explore whether patterns we identify are conserved across evolution.

Outliers are described in the methods section "Using single-cell RNA-seq to resolve parasite populations"

High-throughput data was uploaded to European Nucleotide Archive (accession ERP021229) and ArrayExpress (accession E-ERAD-611) and is stated in the methods section "Code and Data availability"

Statistical reporting

- Statistical analysis methods should be described and justified
- Raw data should be presented in figures whenever informative to do so (typically when N per group is less than 10)
- For each experiment, you should identify the statistical tests used, exact values of N, definitions of center, methods of multiple test correction, and dispersion and precision measures (e.g., mean, median, SD, SEM, confidence intervals; and, for the major substantive results, a measure of effect size (e.g., Pearson's r, Cohen's d)
- Report exact p-values wherever possible alongside the summary statistics and 95% confidence intervals. These should be reported for all key questions and not only when the p-value is less than 0.05.

Please outline where this information can be found within the submission (e.g., sections or figure legends), or explain why this information doesn't apply to your submission:

Protocol evaluation (details in Figure 1 legend)

Clustering (Materials and Methods, "Determining parasite life cycle stages using bulk reference data and clustering")

Variable expression (Materials and Methods, "Assessment of gene expression variation during asexual maturation" and "Determining gene expression variability within different cell types" and "Correction for cell-cycle using scLVM")

GO term analysis (Materials and Methods, "Determining gene expression variability within different cell types")

Transcript half-life analysis (Material and Methods, "Correction for cell-cycle using scLVM")

Co-expression (Figure 4 - figure supplement 4, Figure 6 - figure supplement 2)

Gene family enrichment (Materials and Methods, "Determining gene expression variability within different cell types")

(For large datasets, or papers with a very large number of statistical tests, you may upload a single table file with tests, Ns, etc., with reference to sections in the manuscript.)

Group allocation

- Indicate how samples were allocated into experimental groups (in the case of clinical studies, please specify allocation to treatment method); if randomization was used, please also state if restricted randomization was applied
- Indicate if masking was used during group allocation, data collection and/or data analysis

Please outline where this information can be found within the submission (e.g., sections or figure legends), or explain why this information doesn't apply to your submission:

Group allocation was not relevant to our work. No masking of data was necessary

Additional data files ("source data")

- We encourage you to upload relevant additional data files, such as numerical data that are represented as a graph in a figure, or as a summary table
- Where provided, these should be in the most useful format, and they can be uploaded as "Source data" files linked to a main figure or table
- Include model definition files including the full list of parameters used
- Include code used for data analysis (e.g., R, MatLab)
- Avoid stating that data files are "available upon request"

Please indicate the figures or tables for which source data files have been provided:

Supplementary Data files 1-4 contain lists of genes identified in each analysis and full lists of Gene Ontology terms enriched in these lists where appropriate.

Supplementary Data files 5 and 6 contain the raw count data and meta data for our datasets. We have also set up a GitHub repository containing these data, along with code to analyse it:

https://github.com/adamjamesreid/Plasmodium-single-cell-RNA-seq
